# Silver Nanowires Inks for Flexible Circuit on Photographic Paper Substrate

**DOI:** 10.3390/mi10010022

**Published:** 2018-12-29

**Authors:** Xing Yang, Dexi Du, Yuehui Wang, Yuzhen Zhao

**Affiliations:** 1 School of Materials and Energy, University of Electronic Science and Technology of China, Chengdu 610054, China; shirleywyh@126.com (X.Y.); dudexi_work@foxmail.com (D.D.); 2 Department of Materials and Food, University of Electronic Science and Technology of China Zhongshan Institute, Zhongshan 528402, China; 3 Department of Materials Science and Engineering, Tsinghua University, Beijing 100084, China; zhaoyz@mail.tsinghua.edu.cn

**Keywords:** silver nanowires inks, flexible circuit, photographic paper, sheet resistance

## Abstract

Silver nanowires (AgNWs) have inspired many research interests due to their better properties in optical, electric, and flexible applications. One such exploitable use is as the electrical conductive fillers for print electronics. In this paper, AgNWs with mean a diameter of 80 nm and mean length of 13.49 μm were synthesized using the polyol solvothermal method. A sonication-induced scission process was used to obtain AgNWs with a length range of 7.64–11.21 μm. Further AgNWs inks were prepared with the as-synthesized AgNWs as conductive fillers in anhydrous ethanol. The conductive inks were coated on resin coated photographic paper substrate using the knife coating process and dried at room temperature. The effects of the number of layers of AgNWs coating, the concentration of AgNWs, and the length of AgNWs on the microstructure and electrical properties of samples were investigated by scanning electron microscopy and using the four-point probe method. The results show that the conductivity of the AgNWs coating increases with the increase in the number of layers in the AgNWs coating, concentration and length of the AgNWs.

## 1. Introduction

Paper-based electronic devices have been judged by researchers to hold great promise as an environmentally friendly substrate for flexible electronics due to their inexpensive and common availability worldwide for information storage and packaging [1,2,3]. In recent years, paper-based electronic devices such as “smart paper” were applied to disposable health industry point-of-care bedside [4,5,6,7]. The treatment temperature of paper-based electronic devices must be low because they cannot bear temperatures above 150 °C. As one of the most important parts of the paper-based electronic devices, flexible electrodes are required to be sintered at low temperatures [8,9]. According to our previous results, silver nanowires (AgNWs) in conductive networks can formed at room temperature [10]. 

With the development of flexible electronics, AgNWs have stimulated wide attention due to their unique physical and chemical properties, such as excellent electrical and thermal conductivity, malleability, and chemical stability [8,9,10,11,12]. Notable is the fact that AgNWs can form networks with better mechanical properties, facilitating development of foldable sensors with electrodes capable of withstanding extremely small bending radii without compromising electrical properties [10,11,12,13,14]. AgNWs are now considered as one of the most promising candidate materials to replace indium tin oxide [13,14,15,16].

The properties of silver nanostructures are closely related to morphology and size of AgNWs [17,18,19,20]. Constructing uniformly connected networks of AgNWs with property size is critical for improving the conductivity of electrodes composed of AgNWs and maximizing the potential of AgNWs networks [16,17,18,19,20,21]. However, the preparation of AgNWs with a controllable structure has always been a difficult problem. In the past few years, we have been working on investigating the controllable structure, performance and applications of AgNWs [10,11,21,22,23,24,25]. Bergin and coworkers have recently reported that the transmittance of AgNW film with AgNWs of a given diameter linearly rely on area coverage and do not rely on the length of AgNWs, and that the decrease of AgNWs diameter improves optoelectronic performance only for AgNWs with less than 20 nm in diameter [26]. Anoshkin and coworkers reported that the position of main peaks and the area of the minimal optical absorbance spectra of AgNW layers in the range of 300–700 nm strongly depends on the AgNWs diameter, and three times longer AgNWs gave only about 10% increase of optical transmittance with the same resistivity [27,28].

To realize the marketization and scale of flexible electronics, it is necessary to improve the manufacturing technology of flexible circuits. Conductive inks are the key to achieve flexible circuits. The conductive inks are coated on flexible substrates, such as fabric, paper, Polyethylene terephthalate (PET) and polyimide (PI) by silkscreen printing, inkjet printing, spraying, screen, knife-over-edge, spray coating, gravure, and slot die, to make flexible conductive pathways or transparent conductive films, which are then applied to flexible electronic products [1,2,3,4,5,29]. 

Herein, we prepared AgNWs and obtained AgNWs with the different lengths by the sonication-induced scission method, and further the AgNWs inks were prepared with as-synthesized AgNWs as conductive fillers in anhydrous ethanol. The inks were printed on photographic paper substrate and the effects of the number of AgNWs coating and concentration and size of AgNWs on the microstructure and electrical properties of samples sintered at room temperature were discussed.

## 2. Materials and Methods 

Silver nitrate (≥ 99.8%) was purchased from Guangdong Guanghua Chemical Reagent Co., Ltd. Guangdong, China; poly(vinylprrolidone) (PVP, K30, Mw ≈ 10,000) was purchased from Guoyao Group Chemical Reagent Co., Ltd., Shanghai, China; ferric chloride hexahydrat (≥ 99.5%) was purchased from Chengdu Kelong Chemical Co., Ltd., Chengdu, China; ethylene glycol EG, ≥ 99.7%) and ethanol absolute (≥ 99.7%) were purchased from Tianjin Yongda Chemical Co., Ltd. Tianjin, China; resin coated (RC) photographic papers were purchased from Oracle Technology Co., Ltd. Shenzhen, China. All the chemicals were used as received.

AgNWs were grown via polyol solvothermal method. FeCl_3_ (0.3 mmol·L^−1^) was dissolved in 40 mL EG (0.17 mol·L^−1^) PVP and AgNO_3_ (0.1 mol·L^−1^) were dissolved into 40 mL of EG in turn. Then FeCl_3_-EG solution was dropped into the mixed solution of PVP and AgNO_3_ with a syringe. Please note that the mixed solution was not stirred during the dropping process, and was immediately transferred the into the reaction kettle after the completion of dropping. The reaction kettle was placed in an oven at 160 °C for 3 h. After the completion of reaction, the sample was removed and cooled down to room temperature. Then, acetone and ethanol were used to wash the product. Each washing process was repeated 3 times to remove the extra solvent and chemical agents (PVP and other reactants). The AgNWs were re-dispersed in ethanol absolute.

For ultrasonication treatment, 10.80 mg·mL^−1^ AgNWs solution was put into the beaker filled anhydrous ethanol and sequentially underwent ultrasonication for 1–5 h at a power of 120 W. The ultrasonication was carried out with a bath type sonicator (JP-120ST, 0–600 W, 28/40 kHz, Shenzhen Jiemeng Cleaning Equipment Co., Ltd., Shenzhen, China). AgNWs inks were prepared with as-synthesized AgNWs as conductive fillers in anhydrous ethanol. The stability of the dispersion of AgNWs inks was not good enough. AgNWs settled after being kept at room temperature for 7–15 days or so. However, AgNWs inks have good redispersibility which is usually achieved by ultrasonic stirring.

The inks were coated on photographic paper substrate using the knife coating process. The effects of the number of layers of AgNW coating, the concentration of AgNW ink, and the length of AgNWs on the microstructures and electrical properties of samples dried at room temperature were investigated. 

A scanning electron microscope (SEM, zeiss sigma 500, Carl Zeiss, Oberkochen, Germany) was used to characterize the microstructure of AgNWs. The lengths of individual silver nanowires in the images were measured manually according to an image processing software (ImageJ Software. version 1.46, developed by National Institutes of Health, Bethesda, MA, USA). X-ray diffraction (XRD, DIFFRRACTOMETER, Rigaku Co. Ltd., Tokyo, Japan) was used to measure the phase structures. The sheet resistance of the AgNW film was measured using a 4-point probe instrument ST2253 (Suzhou Jingge Electronics Co., Ltd., Suzhou, China).

## 3. Results

**Figure 1:**
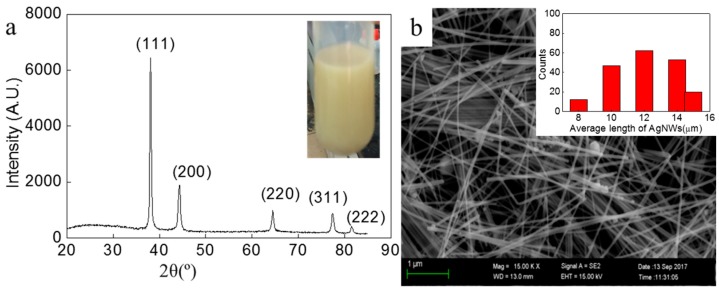
XRD and SEM of as-synthesized silver nanowires (AgNWs). The inserted photo in (**a**) is AgNWs solution without purification. The inserted image in (**b**) is the length distribution of AgNWs.

Figure 1 shows the XRD and SEM image of the purified AgNWs. The inserted photo in Figure 1a is the non- purified AgNW solution. The inserted image in Figure 1b is the length distribution of AgNWs. There are five peaks at 2θ = 38.12°, 44.32°, 65.54°, 77.40°, and 81.56°, corresponding to diffraction from (111), (200), (220), (311) and (222) planes of the face centered-cubic silver crystals, no other phases were detected. The lattice constant was calculated from these XRD patterns according to the spacing distance d_g_ of the (111) plane. The equation is as follows:

1/d^2^_g_ = (h^2^ + k^2^ + l^2^)/α (1)

α = 4.0837 Å, which is close to the reported date of α= 4.0862 Å, JCPDS file 04-0783 [16]. It indicates that a high purity of AgNWs were synthesized. Meanwhile, the peak intensity ratio of (111)/(200) for AgNWs is much higher than that for the standard value, implying the enrichment of (111) crystalline planes of the AgNWs. It can be observed that the length distribution of AgNWs from 8 μm to 15μm, and mean diameter and length are 80 nm and 13.49 μm, respectively (Figure 1b).

**Figure 2:**
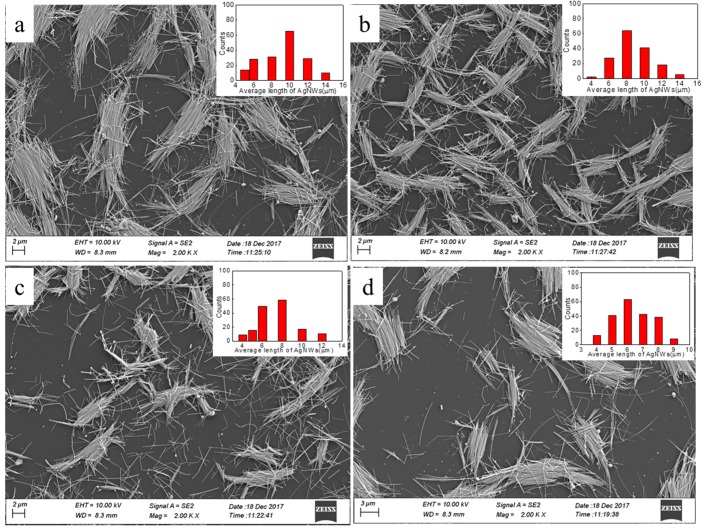
SEM images of AgNWs treated with ultrasonic power 120 W and different ultrasonic time. Ultrasonic time: (**a**) 1 h, (**b**) 2 h, (**c**) 4 h, (**d**) 5 h. The inserted images are the length distribution.

Sonication is widely applied to disperse materials in liquid media due to the ultrahigh shear rate attained during cavitation events [30,31,32]. However, sonication can also induce the scission of the materials that are imploding cavitation bubbles. Sonication-induced scission is often used to solve the problem of scission in fiber-like structures with a high aspect ratio, including the exfoliation and scission of carbon nanotubes [30,31,32]. Here, in order to obtain AgNWs with different lengths, we used a sonication-induced scission process to fracture the AgNWs. Figure 2 shows SEM images of AgNWs (Figure 2a–d) using sonication-induced scission for 1, 2, 4, and 5 h. The inserted images are the length distribution. The distribution of the lengths of the as-synthesized AgNWs allows the average length of AgNWs to be calculated. After being treated by the ultrasonication-induced scission process for 1, 2, 4, and 5 h, the mean lengths of AgNWs are approximately 11.21, 10.8, 8.66, and 7.64 μm, respectively, and the diameter does not change. It is clear that the mean length of the AgNWs decreases as the sonication-induced scission time increases, which is attributed to sonication energy [30,31]. 

**Figure 3:**
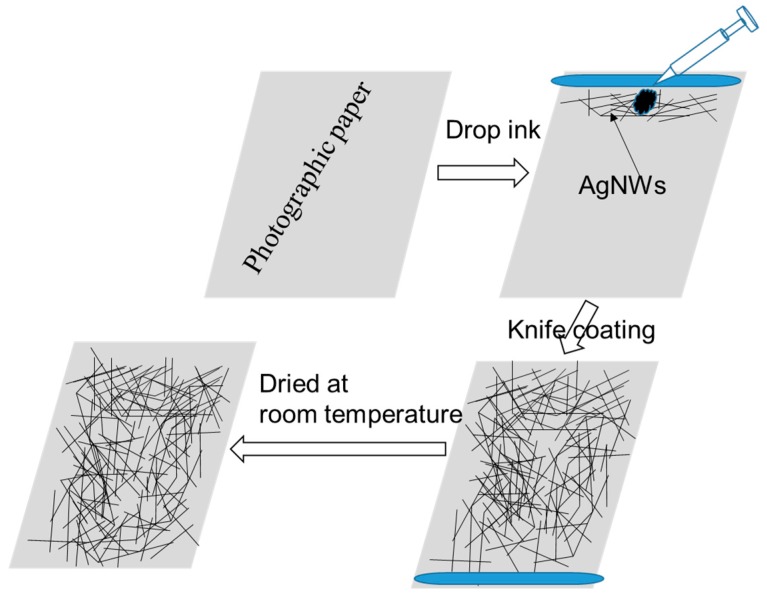
Schematic diagram for the steps applied to fabricate the AgNW coating.

With RC photographic paper as a substrate, the AgNWs coating was prepared by the knife coating process and heat treated at the room temperature. Schematic diagram for the steps applied to fabricate AgNW coating on the photographic paper by knife coating process was shown in Figure 3.

**Figure 4:**
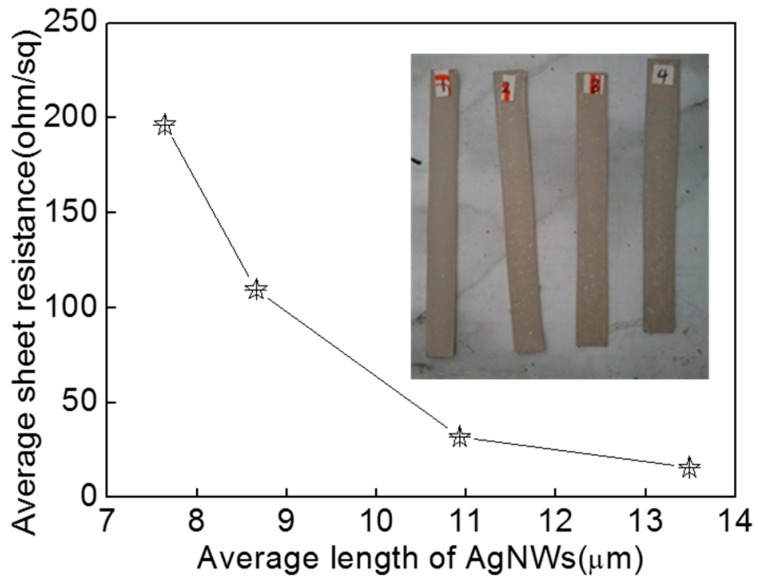
Relationship between average sheet resistance of AgNW coating and length of AgNWs. The inserted image is a photo of samples.

As we all know, the properties of AgNW coating are strongly dependent on the morphology, size and distribution of AgNWs on the substrate [10,11,12,13,14,15]. Here, we obtained AgNWs with different lengths (13.49, 11.21, 10.93, 8.66, 7.64 μm) by sonication-induced scission process and AgNW inks (0.1 mL, 10.80 mg·mL^−1^) were dropped and knife coated the surface of RC photographic paper and dried at the room temperature for 15 min. Figure 4 shows the relationship between the average sheet resistance of AgNW coating and the length of AgNWs. The photos of samples were inserted into Figure 4. We can see the color of AgNW coating is gray with the increase of AgNW length, and the sheet resistance of the coating obviously reduces. When the length of AgNWs is 13.49 μm, the sheet resistance is 15.68 Ω·sq^−1^. When the length of AgNWs is 7.64 μm, the sheet resistance is 196.4 Ω·sq^−1^. The sheet resistance increases by 12.5 times. At the conditions of the same concentration and diameter, the shorter the AgNWs are, the more AgNWs there are. Not surprisingly, the contact resistance among the AgNWs at the same coverage area increases with the increase in the number of AgNWs, leading to a sheet resistance increase.

**Figure 5:**
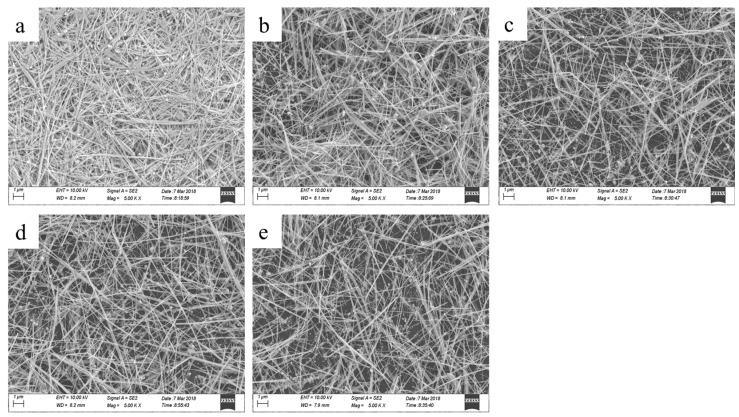
SEM images of the coatings of AgNWs with different lengths, (**a**) 7.64, (**b**) 8.66, (**c**) 10.93, (**d**) 11.21, (**e**) 13.49 μm, respectively.

Figure 5 shows SEM images of coatings of AgNWs with different lengths, from Figure 5a–e is 7.64, 8.66, 10.93, 11.21, and 13.49 μm, respectively. We can observe that the thickness of AgNWs coating and the overlaps among the AgNWs become reduced with the increase of the length of the AgNWs. The densification of the AgNWs coating decreases with the decrease of the length of AgNWs. This is due to the reduction in the number of AgNWs.

**Figure 6:**
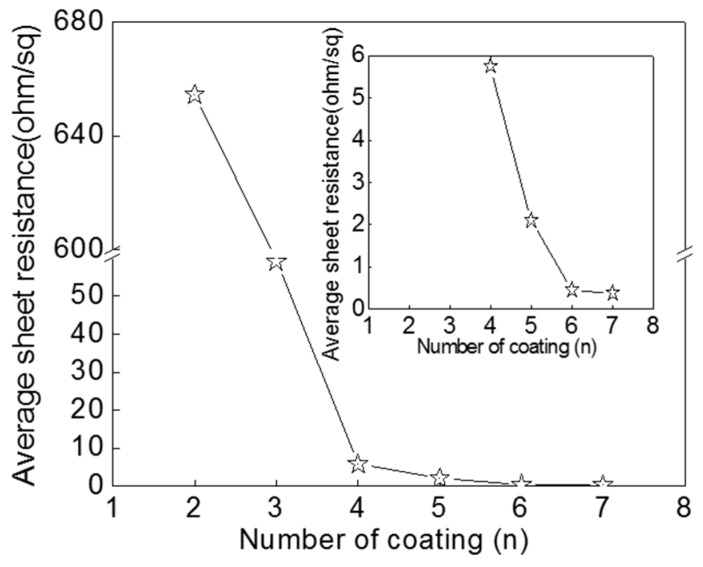
Relationship between average sheet resistance of AgNWs coating and the number of coating layers. The local enlarged image is inserted.

Once the effect of the length of AgNWs on the conductivity of the coating is understood, it is necessary to study the effect of the deposition density of the AgNWs on the conductivity of coating. In order to ascertain the deposition density of the AgNWs dependence, we fabricated the AgNWs (13.49 μm) coatings with different layers. To prepare for multi-layer coating, each sample coated layer is put at room temperature for 15 min, and then coated with the next layer. The concentration of AgNW ink (0.1 mL) is 10.80 mg·mL^−1^. Figure 6 shows relationship between the average sheet resistance of AgNW coating dried at room temperature and the number of coating layers. Inserted is the local enlarged image. During the experiment, we found that AgNWs coating was not as conductive as one coated layer. The average sheet resistance of AgNWs coatings from one layer to five layers, is 654.5, 59.05, 5.87, 2.26 Ω·sq^−1^, respectively. When the layers of coating increased to six the sheet resistance of AgNW coating reduced to 0.45 Ω·sq^−1^. After that, the sheet resistance of AgNWs coating slightly decreased when coating layers were increased to seven. The formation of electrical conduction paths in AgNW coating is considered to contact among the AgNWs. The contact resistance at the interfaces between AgNWs is considered to be strongly influenced by the contact area. The more AgNWs, the more conductive paths are easily formed, which promotes conductivity. However, when the conductive paths in the coating are well formed, the increase of the number of AgNWs coating layers only slightly reduces contact resistance [11,17]. As the conductive material, AgNWs are likely easy to form conductive networks by overlapping each other due to the two-dimensional wire structure.

**Figure 7:**
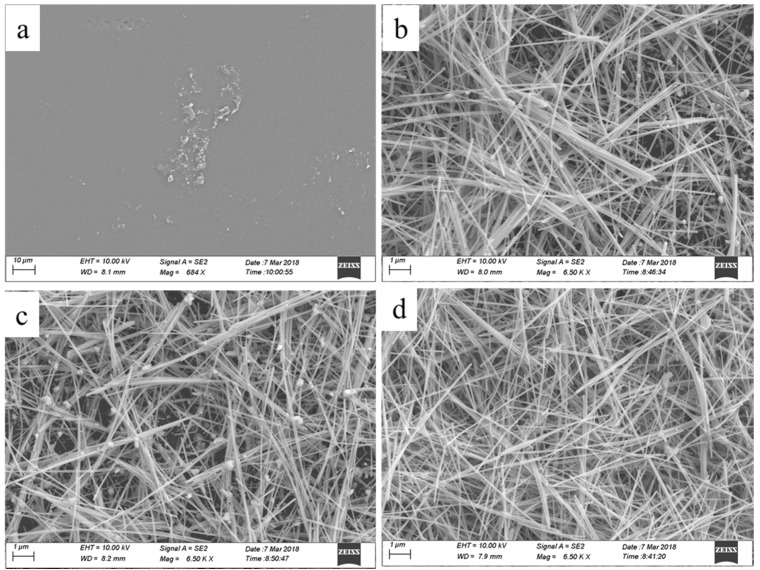
SEM images of AgNWs coatings with different coating layers, (**a**) pure photographic paper, (**b**) 2, (**c**) 4, (**d**) 7 coating layers, respectively.

In order to further analysis of the effect of the number of coating layer on the sheet resistance of AgNWs coating, we observed the microstructures of the samples, as shown in Figure 6, in which Figure 7a–d is the pure photographic paper, 2, 4, and 7 coating layers, respectively. It is clear that with the increase of the number of coating layer, the AgNWs coatings become dense.

**Figure 8:**
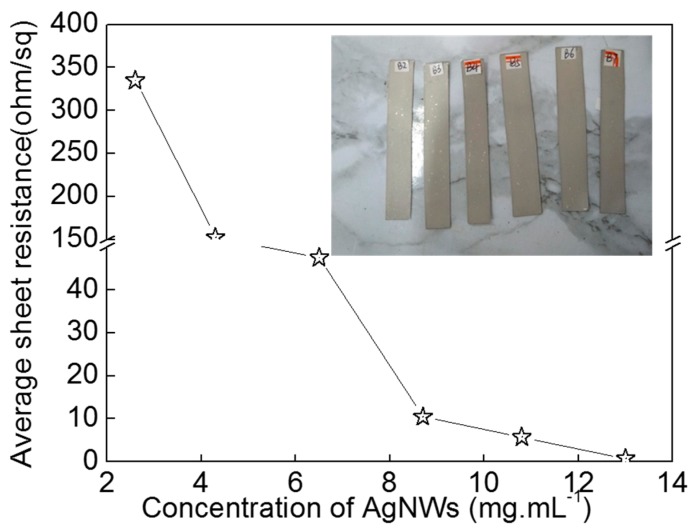
Relationship between average sheet resistance of AgNWs coating and the concentration of AgNWs ink. The inserted image is a photo of the samples.

In order to analyze of the effect of the number of AgNWs on the conductivity of the coating, we prepared the coatings with different AgNW ink concentrations (13.20, 10.80, 8.70, 6.51. 4.32, and 2.65 mg·mL^−1^) and four coating layers, dried at room temperature for 15 min, as shown in Figure 8. The inserted image is a photo of the samples. It is clear that the sheet resistance of AgNWs coating decreases with the increase of concentration of AgNW ink. When the concentration of AgNW ink is 13.20 mg·mL^−1^, the sheet resistance of AgNW coating is 0.77 Ω·sq^−1^, which is close to that of the sample prepared with 10.80 mg·mL^−1^ AgNWs ink and six coating layers (Figure 5). It is indicated that the good conductive coating can be obtained with a high concentration ink by a simple preparation process. However, it is not good for AgNW ink with higher concentration because AgNWs are easy to deposit in the condition of high concentration. When the concentration of AgNWs ink reduces to 2.65 mg·mL^−1^, the sheet resistance of AgNW coating increases to 334.90 Ω·sq^−1^. The more AgNWs that are are added to a coating achieved percolation, the more effective conductive paths are formed, therefore the conductivity of the AgNWs coating will be improved [11,15,16,17,18]. 

**Figure 9:**
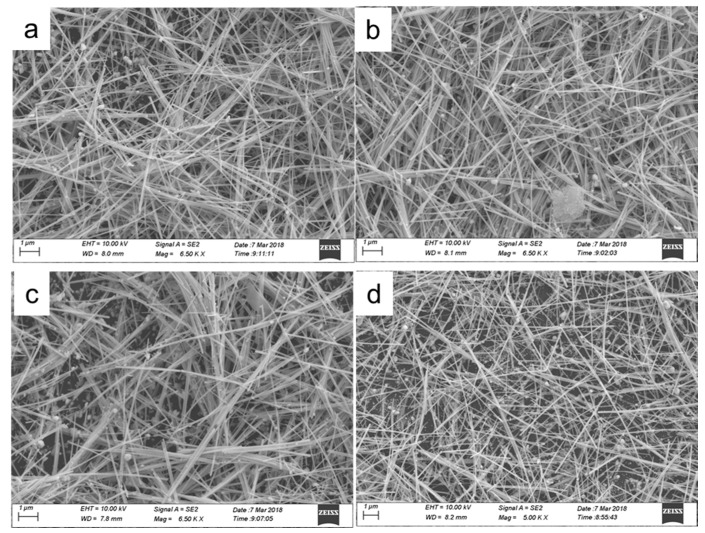
SEM images of coating with the different concentrations of AgNWs, (**a**) 10.8, (**b**) 8.70, (**c**) 4.30, (**d**) 2.61 mol·L^−1^, respectively.

The microstructures of AgNW coating with different concentrations, as shown in Figure 9, in Figure 9a–d is 10.80, 8.70, 4.30, and 2.61 mol·L^−1^, respectively. It is clear that with the increase of concentration of AgNW ink, the thickness of coatings also increases.

**Figure 10:**
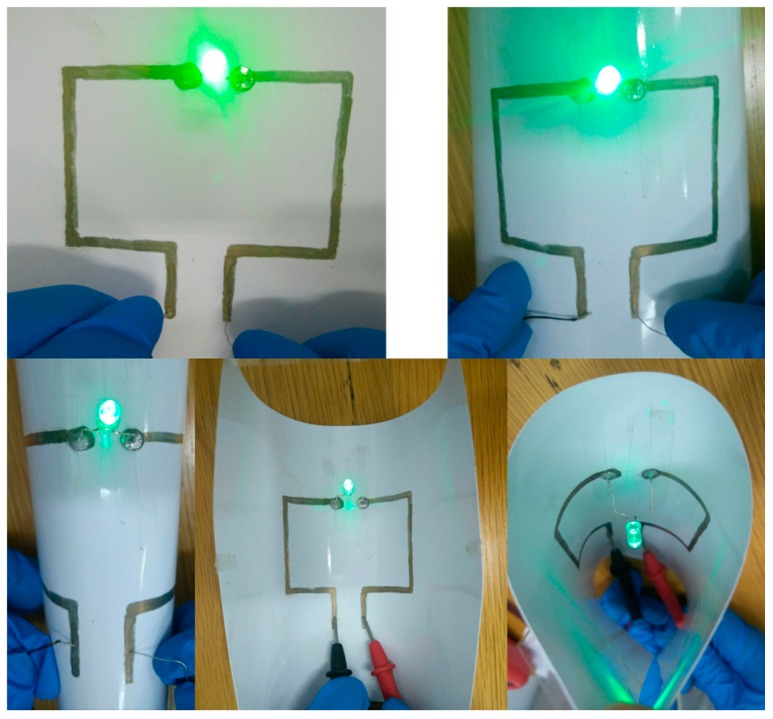
Optical images of light emitting devices with the AgNW conductive circuit on photographic paper.

As discussed above, the AgNW inks are viable in the application of the flexible devices. Here, the potential application of the AgNWs ink by fabricating an LED device on the AgNW electrodes was measured (Figure 10). One 0.5-W LED lamp was fixed onto the surface of the AgNW electrode with conductive adhesive on the photographic paper substrate. In can be seen that the LED lamp was lit whether the photo paper was flat or bent, indicating the good conductivity and mechanical behavior of the AgNW coating.

## 4. Conclusions

AgNWs with a mean diameter of 80 nm and mean length of 13.49 μm were synthesized using the polyol solvothermal method. Then sonication-induced scission process was used to obtain AgNWs with a length range of 7.64–11.21 μm, and further the AgNWs inks were prepared with as-synthesized silver nanowires as conductive fillers in anhydrous ethanol. The conductive inks were coated on photographic paper substrate using the knife coating process and dried at room temperature for 15 min. We demonstrate that the conductivity and densification of AgNW coating increases with the increase in the number of AgNW coating layers, concentration and length of the AgNWs. When the length of AgNWs is 13.49 μm, the sheet resistance is 15.68 Ω·sq^−1^. When the length of AgNWs reduces to 7.64 μm, the sheet resistance increases to 196.4 Ω·sq^−1^. The sheet resistance increases by 12.5 times. When the concentration of AgNW ink is 13.20 mg·mL^−1^, the sheet resistance of AgNW coating with four layers is 0.77 Ω·sq^−1^, which is close to that of the sample that was prepared with 10.80 mg·mL^−1^ AgNW ink and six coating layers.
